# Residents in a Remote Island Having Family Members in Distant Areas Showed Higher Preference for Place of End-of-Life Care: The Ajishima Study

**DOI:** 10.31662/jmaj.2019-0026

**Published:** 2020-04-10

**Authors:** Kemmyo Sugiyama, Toru Tsuboya, Taketoshi Okita, Naho Tsuchiya, Kunio Tarasawa, Tomoaki Ogata, Shintaro Yanaka, Akio Tomoda

**Affiliations:** 1 Liaison Center for Innovative Dentistry, Tohoku University Graduate School of Dentistry, Sendai, Japan; 2 Department of Internal Medicine, Amishou Clinic, Ishinomaki, Japan; 3 Department of Medical Ethics, Tohoku University Graduate School of Medicine, Sendai, Japan; 4 Department of Preventive Medicine and Epidemiology, Tohoku Medical Megabank Organization, Tohoku University, Sendai, Japan; 5 Department of Health Administration and Policy, Tohoku University Graduate School of Medicine, Sendai, Japan; 6 Division of Health Administration and Policy, Tohoku Medical and pharmaceutical University, Faculty of Medicine, Sendai, Japan

**Keywords:** advance care planning, end-of-life care, family, remote medicine

## Abstract

**Introduction:**

To investigate the proportion of those having preferred place for end-of-life care among residents in a remote island and its association with family composition.

**Methods:**

Cross-sectional questionnaire survey was conducted in Ajishima, an island 23 km away from the coast of Ishinomaki City, northeast of Japan. Between October 2017 and February 2018, the questionnaire was distributed to 288 eligible residents and 113 valid responses were analyzed. Primary outcome was whether the subjects had preferred place for end-of-life care. The explanatory variable was family composition defined as whether having family members inside or outside the island [none (In−/Out−), only inside the island (In+/Out−), only outside the island (In−/Out+), and both inside and outside (In+/Out+)]. Poisson regression analysis was used to calculate the prevalence ratios (PRs) and 95% confidence intervals (CIs) of showing preferred place in each group.

**Results:**

The proportion of those having preferred place for end-of-life care was 72.6% in total. This rate significantly differed across family composition groups: 67.6%, 40.0%, and 82.9% for In+/Out+, In+/Out−, and In−/Out+ groups, respectively. The PR (95%CI) of having preferred place was 0.66 (0.33, 1.36) and 1.26 (1.01, 1.56) for In+/Out− and In−/Out+ groups, respectively, compared with In+/Out+ group.

**Conclusions:**

This study showed that significantly higher preference for place of end-of-life care was seen among residents who had family members only outside the island compared with those who had families both inside and outside. Health care professionals should consider family compositions when initiating end-of-life discussion to residents in remote areas.

## Introduction

Every individual has the right to end life with dignity ^(1)^. This can be maintained when one’s preferences toward end of life are fulfilled ^(2), (3)^. Among these preferences, place for end-of-life care had become a major issue especially in ageing societies ^(4), (5), (6)^. However, data generally show that there is a huge gap between individuals’ preferences ^(7)^ and actual place of death ^(8)^. In Japan, one of the leading ageing societies throughout the world, a nationwide survey reported that approximately 75% of the respondents preferred to die at home ^(9)^, whereas the statistics showed that almost 70% of the whole population died at hospitals ^(10)^. To fill this gap, it is essential to promote advance directive (AD). AD is a written document that indicates an individual’s choices about medical treatment such as life-sustaining treatments (cardiopulmonary resuscitation, mechanical ventilation, hemodialysis, antibiotics, artificial nutrition or hydration, etc.) in advance of severe medical condition with difficulty of providing one’s thought due to loss of consciousness or mental dysfunction. Here, we consider that preference of being transferred to hospitals at end-of-life stage is also one of the important factors of AD ^(11)^. AD had gained importance and become legislated in several countries ^(11), (12), (13, (14)^. However, AD had not been widely conducted among the nations as expected ^(14)^. Despite that whether an individual possesses AD or not, it is still important for one to have an opportunity of giving thoughts, to communicate, and being heard about future plans on preferred treatment and care at end-of-life stage. This process is called advance care planning (ACP). Promotions of ACP are held worldwide ^(15)^ including Japan ^(16)^.

In general, people in remote areas are highly restricted in access to medical, home care, or welfare service ^(17)^. Thus, ACP on where to receive end-of-life care is essential. However, it has rarely been reported to how much extent residents in remote areas showed their preferred places. Therefore, we conducted a cross-sectional study in a remote island to investigate 1) the proportion of those who have preferred places for end-of-life care and 2) the association between family composition and having preferred places

## Materials and Methods

### Demographics of the study area

**Figure 1.**
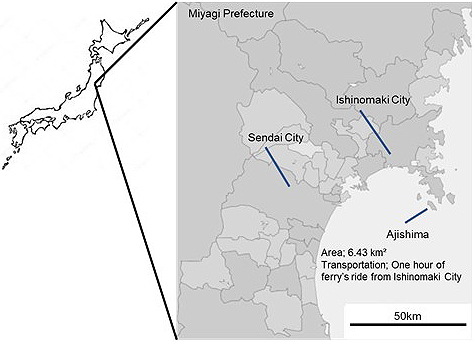
Geographic of Ajishima.

This study was conducted in Ajishima, an island (6.43 km^2^) located approximately 23 km away (a 1 h ride of ferry boat) from the coast of Ishinomaki City, a northeastern city in Japan (Figure 1). The population was once approximately 3,000 in the 1950s but extremely reduced to 355 in 2017, with 71.2% of the population being ≥65 years of age ^(18)^. Amishou Clinic is the single clinic in the island, privately established in 1999 after the preceding public one had closed. Only outpatients are seen. Until the year 2012, there was only one physician commuting to the clinic every day. At present, physicians commute to the clinic during weekday daytimes. During the weekends, physicians stay in the island. Eventually, there are no physicians in the island during weekday nights except for nurses residing in the island. Fortunately, there has been only one case of emergency transportation during night in the past 20 years. However, it involves risks and costs to transfer emergency patients across the ocean by chartered private boats during late evenings ($1,500 per one ride). Strikingly, no transportation is prepared during nights.

### Data collection

**Figure 2.**
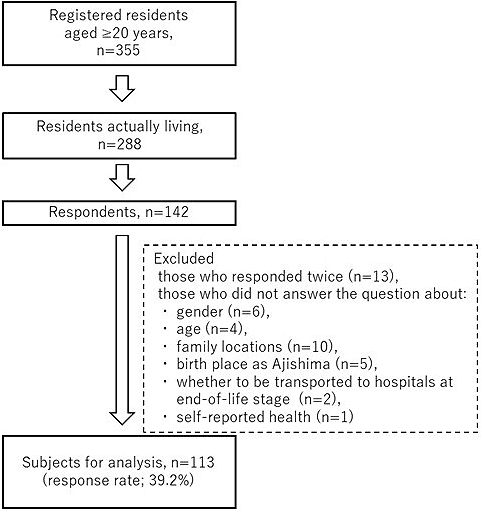
Flow chart of Ajishima Study.

Of the 355 registered population, 288 individuals were clarified to live in the island within the last 6 months. On October 28 and 29, questionnaires were distributed to participants aged ≥65 years and collected directly at Amishou Clinic. On February 11, 2018, same questionnaires were distributed to those aged <65 years at two public halls in the island. Eventually, a total of 142 individuals responded. After excluding those who responded twice (n = 13) and those who did not answer the question about gender (n = 6), age (n = 4), family locations (n = 10), whether given birth in Ajishima (n = 5), whether to be transported to hospitals at end-of-life stage (n = 2), and self-reported health (n = 1), 113 respondents with complete responses were eligible for analysis (39.2% of the total 288 residents; Figure 2).

The questionnaire inquired about whether having a preferred place for end-of-life care at the date of the survey, which was the primary outcome. This was asked through a question, “Do you desire to be transferred outside the island and be admitted to a hospital in the city for end-of-life care when you are senile.” The prepared answers were “yes,” “no,” and “have not decided.” For the analysis, we regarded the two former answers “yes” or “no” as Preference+, and “have not decided” as Preference−. We designed to inquire only this question because this questionnaire itself could not be considered as an official AD from clinical and ethical viewpoints.

The explanatory variable was “family composition” defined as whether they had family member inside or outside the island. Presence of family has been a major factor of dying at home ^(19), (20)^. However, there are more various types of family composition in rural areas, such as settings of families living inside or outside the residential areas. The impact of such family composition on having ACP is expected to be considerable, though seldom studies had investigated this issue before.

Variable for family composition was created by combining two questions: “Do you have a family living inside the island?” and “Do you have a family living outside the island?” Thus, we made four categories of families living inside and/or outside the island: yes and yes (In+/Out+), yes and no (In+/Out−), no and yes (In−/Out+), and no and no (In−/Out−), respectively. As a result, there were no cases in the group In−/Out−. Therefore, we used the other three categories for analysis.

Other items such as gender, age groups according to quintiles (<67, 68–75, 76–81, 82–85, and ≥86 years), whether being born in Ajishima, and self-reported health (very good, good, bad, and very bad) ^(21)^ were also inquired. These were considered as covariates that were reported to be associated with home death ^(17), (22), (23), (24)^. For the multivariate analysis, the answers for self-reported health were combined into two categories (health and unhealthy) because of the small number of cases in “very good” and “very bad” categories.

### Statistical analysis

We first counted the frequency of Preference+ and the other baseline characteristics. We also tested the statistical differences among the location types of families by using the chi-square test. Then, we used Poisson regression analysis to calculate the prevalence ratios (PRs) and 95% confidence intervals (CIs) of Preference+ for the In+/Out− and In−/Out+ groups, compared with the In+/Out+ group. Poisson regression model was used because it provides less biased estimates than logistic regression when the prevalence is high ^(25)^. In addition, we conducted similar multivariate analyses stratified by gender and birthplace, expecting that the percentage of these factors differed among the three family living arrangements. All *p*-values were two-tailed, considering p < 0.05 as statistically significant. All analyses were conducted by using SPSS 23.0 for Windows (SPSS Inc., Chicago, IL, USA).

This study was approved by the Ethics Committee of the Tohoku University Graduate School of Dentistry (code: 2017-3-12). We considered each participant’s response to the questionnaire as their consent to participate in the survey. 

## Results

Table 1 shows the frequency of Preference+ for each family composition group. First, the percentage of those who answered “yes” to the question “Do you desire to be admitted outside the island for end-of-life care?” was 32.3%, 22.2%, and 28.2% among the In+/Out+, In+/Out−, and In−/Out+ groups, respectively. The percentage for the answer “no” was 36.9%, 22.2%, and 56.4%, respectively. These answers were combined into Preference+ with the rate being 69.2%, 44.4%, and 84.6% for each family composition group, respectively. In contrast, the percentage of Preference−, meaning “have not decided on preferred place,” was 30.8%, 55.6%, and 15.4% for each group, respectively. The chi-square test showed that there was significant difference among the three groups (*p *= 0.020). Overall, the rate of Preference+ was 72.6% in total.

**Table 1.** Baseline Characteristics According to Family Composition (n = 113).

	Total		Family composition						*p*-value^a^
			In+/Out+		In+/Out−		In−/Out+		
	n = 113		n = 65		n = 9		n = 39		
Preference to be transferred and admitted outside the island at end-of-life, n (%)									0.079
Yes	34	(30.1)	21	(32.3)	2	(22.2)	11	(28.2)	
No	48	(42.5)	24	(36.9)	2	(22.2)	22	(56.4)	
Have Not decided	31	(27.4)	20	(30.8)	5	(55.6)	6	(15.4)	
Ever involved in ACP, n (%)									0.034
Yes	82	(72.6)	45	(69.2)	4	(44.4)	33	(84.6)	
No	31	(27.4)	20	(30.8)	5	(55.6)	6	(15.4)	
Gender, n (%)									0.005
Female	59	(52.2)	29	(44.6)	2	(22.2)	28	(71.8)	
Male	54	(47.8)	36	(55.4)	7	(77.8)	11	(28.2)	
Age group, n (%)									0.085
≤67 years	19	(16.8)	10	(15.4)	1	(11.1)	8	(20.5)	
68–75 years	24	(21.2)	14	(21.5)	4	(44.4)	6	(15.4)	
76–81 years	24	(21.2)	19	(29.2)	1	(11.1)	4	(10.3)	
82–85 years	24	(21.2)	12	(18.5)	3	(33.3)	9	(23.1)	
≥86 years	22	(19.5)	10	(15.4)	0	(0.0)	12	(30.8)	
Born in Ajishima, n (%)									0.821
Yes	82	(72.6)	48	(73.8)	7	(77.8)	27	(69.2)	
No	31	(27.4)	17	(26.2)	2	(22.2)	12	(30.8)	
Self-Reported Health, n (%)									0.102
Very good	8	(7.1)	2	(3.1)	1	(11.1)	5	(12.8)	
Good	76	(67.3)	43	(66.2)	7	(77.8)	26	(66.7)	
Bad	23	(20.4)	18	(27.7)	1	(11.1)	4	(10.3)	
Very Bad	6	(5.3)	2	(3.1)	0	(0.0)	4	(10.3)	

^a^ *P*-value calculated by using a chi-square test.

Table 1 also shows the baseline characteristics according to each group. The In−/Out+ group had higher rate for women (71.8%) with statistical difference among the three family location groups. The In−/Out+ group also had higher rate for those aged ≥86 years (30.8%) and reporting their health to be very bad (10.3%). No statistical difference was observed for both factors among the three groups. No significant difference was also observed in the rate of being born in Ajishima among the groups.

Table 2 shows the results for Poisson regression analysis. Model 1 is crude model showing that the tendency of Preference+ was insignificantly higher in the In−/Out+ group with the PR (95% CI) being 1.22 (0.99–1.51) compared with the In+/Out+ group. On the other hand, the PR was insignificantly lower among the In+/Out− group. Model 2, adjusted for gender and age groups, showed similar results with Model 1. Finally, Model 3, the multivariate-adjusted model, showed that PRs (95% CIs) were 0.66 (0.33, 1.36), 1.26 (1.01, 1.56) for In+/Out− and In−/Out+ groups, respectively: those who had families only outside the island had higher PR of Preference+. No significant results were observed in other factors.

**Table 2.** The Prevalence Ratio (PR) and 95% Confidence Interval (CI) of Having Preferred Place for End-of-Life Care According to Family Composition (n = 113).

	Model 1	Model 2	Model 3
	PR (95% CI)	PR (95% CI)	PR (95% CI)
Family composition			
In+/Out+	(reference）	(reference）	(reference）
In+/Out−	0.64 (0.30, 1.36)	0.66 (0.31, 1.41)	0.66 (0.33, 1.36)
In−/Out+	1.22 (0.99, 1.51)	1.22 (0.98, 1.51)	1.26 (1.01, 1.56)
Gender			
Male		(reference）	(reference）
Female		0.99 (0.78, 1.26)	1.04 (0.81, 1.33)
Age group			
≤67 years		(reference）	(reference）
68–75 years		1.16 (0.70, 1.93)	1.18 (0.72, 1.95)
76–81 years		1.36 (0.87, 2.13)	1.43 (0.92, 2.21)
82–85 years		1.42 (0.91, 2.22)	1.44 (0.92, 2.24)
≥86 years		1.44 (0.93, 2.23)	1.30 (0.83, 2.02)
Born in Ajishima			
Yes			(reference）
No			0.71 (0.52, 0.96)
Self-Reported Health			
Healthy			(reference）
Unhealthy			1.14 (0.89, 1.46)

Table 3 shows the results for similar multivariate analyses stratified by gender and whether born in Ajishima. When stratified by gender, there was no association between family composition and Preference+ among women, whereas the PR was significantly high among men in the In−/Out+ group. However, no significant interaction was observed among both genders. On the other hand, for the stratification of birthplace, there was also a significant PR for those being born in Ajishima and having a family In−/Out+, though the interaction was not significant.

**Table 3.** The Prevalence Ratio (PR) and 95% Confidence Interval (CI) of Having Preferred Place for End-of-Life Care According to Family Composition Stratified by Gender and Birthplace.

	Family composition			*p for interaction*
	In+/Out+	In+/Out−	In−/Out+	
Gender^1^				0.890
Women (n = 59)				
Have ACP, n (%)	22 (75.9)	1 (50.0)	23 (82.1)	
PR (95％ CI)^2^	(reference）	0.70 (0.21, 2.31)	1.13 (0.85, 1.52)	
Men (n = 54)				
Have ACP, n (%)	23 (63.9)	3 (42.9)	10 (90.9)	
PR (95％ CI)^2^	(reference）	0.76 (0.33, 1.75)	1.77 (1.22, 2.57)	
Born in Ajishima^2^				0.284
Yes (n = 82)				
Have ACP, n (%)	35 (72.9)	4 (57.1)	25 (92.6)	
PR (95％ CI)^4^	(reference）	0.82 (0.42, 1.59)	1.26 (1.02, 1.56)	
No (n = 31)				
Have ACP, n (%)	10 (58.8)	0 (0)	8 (66.7)	
PR (95％ CI)^4^	(reference）	NA	1.36 (0.65, 2.85)	

^1^Subjects who had missing information about gender (n = 3) were excluded. ^2^Adjusted for age groups (≤67 years, 68–75 years, 76–81 years, 82–85 years, ≥86 years), whether born in Ajishima (Yes, No, and missing), and self-reported health (healthy and unhealthy). ^3^Subjects who had missing information on whether being born in Ajishima (n = 2) were excluded. ^4^Adjusted for gender, age groups (≤67 years, 68–75 years, 76–81 years, 82–85 years, ≥86 years), and self-reported health (healthy and unhealthy).

## Discussion

It was shown that approximately 70% of the total residents expressed their preferred place for end-of-life care. Furthermore, those who had family outside the island and none inside had significantly higher preference for place of end-of-life care compared with those having family members both inside and outside the island.

This study was conducted in Japan, one of the most ageing societies in the world. More than 1.5 million deaths occur annually in Japan, of which 70% took place in hospitals ^(10)^. If this trend in places for death does not shift from hospitals to homes in the future, it is anticipated that there will be a mismatch of supply and demand in hospitals. In detail, the Japanese government is planning to regulate the present 1.6 million hospital beds ^(26)^ to 1.2 million by 2025 in order to suppress the increasing medical expenses and offer more supply for long-term care instead of medical services ^(27)^. On the contrary, the growing number of elderly people has been anticipated to result in 1.6 million annual deaths in 2025 ^(28)^. Thus, hospital beds will lose its function as providing medical service for treatable patients. Instead, they will be occupied by patients in need of not treatment but end-of-life care. Japan will confront not only this gap between medical demand and supply but also other ageing Organization for Economic Co-operation and Development countries ^(29)^ and those future aging societies in Latin America, Caribbean, and Asia ^(30)^. In order to avoid this gap, AD is essential to accomplish individuals’ desires. Still, AD had not become widespread. The percentage of individuals possessing AD varied from 30% to 70% across countries. Our result showed that the preference was comparatively high (69.2%). We consider that this was largely due to the high ageing rates in the island.

To the best of our knowledge, though there had been a study on the effect of family structure ^(4)^ or marital status ^(31)^, studies on family locations had been rare. Therefore, our results provided new evidence that family location was associated with having end-of-life preferences. Considering that a report in the year 2015 had presumed that population in 70% of the entire Japanese municipalities would decrease ^(28)^, we assume that majority of the families are those moving out of the areas. Therefore, it is likely that family settings in other rural areas will follow the situation in Ajishima in the near future. However, careful interpretation is needed because our data have been obtained from remote islands with limited populations as well as limited transportation facilities. Future studies are required to seek whether our findings would also be observed in non-isolated rural or urban areas.

When discussing the generalizability of our results to other countries, we should consider that whether family members live together throughout life depend on historical, cultural, industrial, or economic settings of that race or country. The US or Northern European elderly people have preferred to live apart from their children after the WWII ^(32)^. In France, Greece, and Italy, the percentage of youth living with their parents has risen ^(33)^. In Japan, baby boom after WWII and the high economic growth between the 1950s and 1970s led younger generations to live away from their parents in the rural areas and form new families in the cities ^(34)^. On the contrary, majority of families live together traditionally in Asian developing countries ^(35)^. These social, cultural, and geographical conditions should also be included in the interpretation of the generalizability of our results.

On the contrary, there was no association between having a family inside the island and preferred place for end of life. This finding is surprising because families living together have more chance in time to discuss end of life. Further qualitative investigation is necessary. Similarly, stratifying analysis showed that being born in Ajishima and have family members only outside the island had higher proportion of preferences (92.6%) compared with those who have family members inside. To our surprise, those who emigrated from other areas had even lower proportion compared with those born in Ajishima. If these immigrants were less familiar with other areas around the island, then they would have been anticipated to select the island as their preferred place at end of life. Further interview is also needed on this issue. If it is merely an issue that they have not experienced end-of-life discussion yet, it is the chance for health care professionals to inform them, which is usually feasible in remote areas.

Our study had several limitations. First, we did not collect information on medical records or disability levels, which were found to be associated with home death ^(36)^. Instead, we alternatively collected self-reported health status, which has been previously reported to correlate with comorbidities ^(37)^ and mortality ^(38)^. Second, the response rates were relatively low, especially for younger adults. Considering that the younger adults have less awareness on end of life, our results for younger adults may have overestimated the rate of having ACP. Third, our results may have been biased because the questionnaires were collected at the clinic. Fourth, we only asked their current thoughts, and we did not ask whether they had discussed with their families. These factors may have impacted our results.

After elucidating the impact of family location, the next step should be on how to promote ACP, considering that legislation on AD had not been effective ^(14)^. Japanese latest medical payment system introduced financial incentives when ACP was facilitated ^(39)^. Future studies are required to evaluate the effects of ACP promotions.

### Conclusion

We conducted a questionnaire survey in a remote island to find that almost 70% of the residents had preferred place for end-of-life care. Moreover, having family members only in distant areas was associated with having such preferred places. It is essential to consider family composition when facilitating ACP, especially in rural areas where medical and long-term care services are limited.

## Article Information

### Conflicts of Interest

None

### Sources of Funding

This work was supported by the Grant-in-Aid of the Miyagi Public Health Association, Japan.

### Acknowledgement

We are grateful to all participants who participated in the Ajishima Study. We are also grateful to the staffs of Ajishima Clinic for supporting this study.

### Author Contributions

KS, TT, TO, NT, KT, TO, SY, AT designed the research, KS conducted the research and collected the data; KS analyzed the data; KS, TT, TO, NT, KT, TO, SY, AT prepared the paper; KS, TT, TO, KT, KT, TO, SY, AT had primary responsibility for final content.

### Approval by Institutional Review Board (IRB)

This study protocol was approved by the Ethics Committee of Tohoku University Graduate School of Dentistry (code: 2017-3-12).
